# Contribution of miRNAs in the Pathogenesis of Breast Cancer

**DOI:** 10.3389/fonc.2021.768949

**Published:** 2021-11-05

**Authors:** Soudeh Ghafouri-Fard, Ali Khanbabapour Sasi, Atefe Abak, Hamed Shoorei, Ali Khoshkar, Mohammad Taheri

**Affiliations:** ^1^ Department of Medical Genetics, School of Medicine, Shahid Beheshti University of Medical Sciences, Tehran, Iran; ^2^ Biochemistry Group, School of Medicine, Golestan University of Medical Science, Gorgan, Iran; ^3^ Men’s Health and Reproductive Health Research Center, Shahid Beheshti University of Medical Sciences, Tehran, Iran; ^4^ Department of Anatomical Sciences, Faculty of Medicine, Birjand University of Medical Sciences, Birjand, Iran; ^5^ Department of Surgery, Loghman Hakim Hospital, Shahid Beheshti University of Medical Sciences, Tehran, Iran; ^6^ Skull Base Research Center, Loghman Hakim Hospital, Shahid Beheshti University of Medical Sciences, Tehran, Iran

**Keywords:** miRNA, microRNA, breast cancer, apoptosis, biomarker

## Abstract

Breast cancer is the most frequently diagnosed cancer among females. Gene expression profiling methods have shown the deregulation of several genes in breast cancer samples and have confirmed the heterogeneous nature of breast cancer at the genomic level. microRNAs (miRNAs) are among the recently appreciated contributors in breast carcinogenic processes. These small-sized transcripts have been shown to partake in breast carcinogenesis through modulation of apoptosis, autophagy, and epithelial–mesenchymal transition. Moreover, they can confer resistance to chemotherapy. Based on the contribution of miRNAs in almost all fundamental aspects of breast carcinogenesis, therapeutic intervention with their expression might affect the course of this disorder. Moreover, the presence of miRNAs in the peripheral blood of patients potentiates these transcripts as tools for non-invasive diagnosis of breast cancer.

## Introduction

Breast cancer is the most frequently diagnosed cancer among females. With approximately 2.3 million new cases, breast cancer accounts for 11.7% of all diagnosed cancers. In terms of cancer-related mortalities, female breast cancer is responsible for 6.9% of mortalities and ranks fifth. Notably, the mortality rate from female breast cancer is significantly higher in developing countries compared with that in developed countries (1). This cancer has been found to be associated with a number of lifestyle and reproductive risk factors, namely, early menarche age, late menopause age, first birth high age, lower period of breastfeeding, hormone replacement therapy after menopause, taking oral contraceptive pills, alcohol intake, and obesity (2). Approximately 5–10% of breast neoplasms are associated with inherited mutations in a number of genes, particularly the BRCA1 and BRCA2 genes (3). In addition, gene expression profiling methods have shown the deregulation of several genes in breast cancer samples and have confirmed the heterogeneous nature of breast cancer at the genomic level (4). More recently, several investigations have reported the dysregulation of microRNAs (miRNAs) in breast cancer samples or plasma samples from these patients in correlation with the functional aspects of tumorigenesis (5–7). miRNAs are produced through a multistep process mediated by two RNase III proteins, namely, Drosha and Dicer (8, 9). These small-sized non-coding transcripts have been found to regulate the expression of a significant proportion of human genes and play fundamental roles in the development of human disorders (10). miRNAs mainly regulate gene expression at post-transcriptional level. Meanwhile, miRNA metabolism and functions are regulated through sophisticated mechanisms (10). Moreover, the expression of miRNA genes is regulated at the transcriptional level through mechanisms similar to the regulatory mechanisms of protein-coding genes. This type of regulation defines the tissue- or developmental stage-specific expression of miRNAs. Most notably, miRNAs can suppress the expression of mRNAs that code factors participating in miRNA biogenesis; thus, they contribute in autoregulatory feedback paths (10). The expression of miRNAs has been reported to be altered in breast cancer samples. As an illustration, recent studies have detected the aberrant expression of miR-221 and miR-222 in breast malignancy (11, 12). In the current review, we describe the impact of miRNAs in breast carcinogenesis and explain their participation in the regulation of apoptosis, autophagy, epithelial–mesenchymal transition (EMT), and resistance to chemotherapy. These processes have important roles in the pathogenesis of cancer. EMT is regarded as a key participant in the invasion and metastasis of cancers. Thus, identifying the main regulators of this process has important implications in cancer treatment. Autophagy has dual roles in cancer progression. Its activation can provide energy and nutrient supplies during the metastatic process, which promotes cell survival in stressful situations (13). In contrast, autophagy can act as a cancer suppressor in the early phase of cancer progression and hinder metastasis through decreasing the expression of important transcription factors for EMT (13). Resistance to apoptotic signals is a key feature in cancer development (14). Moreover, defects in the apoptotic mechanisms enhance malignant transformation and induce the resistance of transformed cells to chemotherapy (14). Finally, resistance to chemotherapy is an important feature gained by tumor cells during tumor evolution, precluding cancer management.

## Regulation of Apoptosis by miRNAs in Breast Cancer

Apoptosis is a coordinated process that happens in physiological and pathological contexts. Cancer is one of the contexts where lack of appropriate cell apoptosis results in the survival of malignant cells. Several pathways are involved in the regulation of apoptosis. Defects can happen at any portion of these pathways, resulting in the malignant transformation of cells, facilitation of tumor metastasis, and induction of resistance to anticancer agents (15). miR-7-5p is an example of miRNAs that regulate the apoptosis of breast cancer cells. This miRNA has been shown to target proteasome activator subunit 3 (REGγ), an important modulator of breast cancer and activator of protein proteolysis. The upregulation of miR-7-5p has led to the suppression of proliferation and induction of cell apoptosis in breast cancer through influencing the expression of REGγ (16). This member of the REG family has an oncogenic function which depends on the proteolysis of p21 and p53 (17, 18). miR-15a and miR-16 are two other miRNAs that regulate the apoptosis of breast cancer cells. Luciferase reporter assay has confirmed the interaction between these miRNAs and 3′ UTR of BMI1 transcript. Both miRNAs could suppress the expression of BMI1 at the transcript and protein levels, resulting in the downregulation of anti-apoptotic protein BCL2 and the upregulation of pro-apoptotic proteins. The forced over-expression of these miRNAs has enhanced the levels of mitochondrial reactive oxygen species (ROS), leading to impairment of mitochondrial membrane potential, release of cytochrome c into the cytosol, and activation of Caspase-3 and Caspase-6/9. These events altogether induce the intrinsic pathway of apoptosis (19). miR-17-5p is another miRNA that has been found to induce apoptosis in breast cancer cells. Notably, the upregulation of miR-17-5p has enhanced the sensitivity of breast cancer cells to paclitaxel-associated cell apoptosis through the modulation of STAT3. Consistent with this finding, the upregulation of STAT3 has reduced the paclitaxel-associated apoptosis of MCF-7 cells. miR-17-5p has been found to enhance apoptosis through upregulating the p53 expression, which was suppressed by STAT3. Therefore, miR-17-5p suppresses STAT3 and upregulates p53 to increase breast cancer cell apoptosis (20).

**Figure 1:**
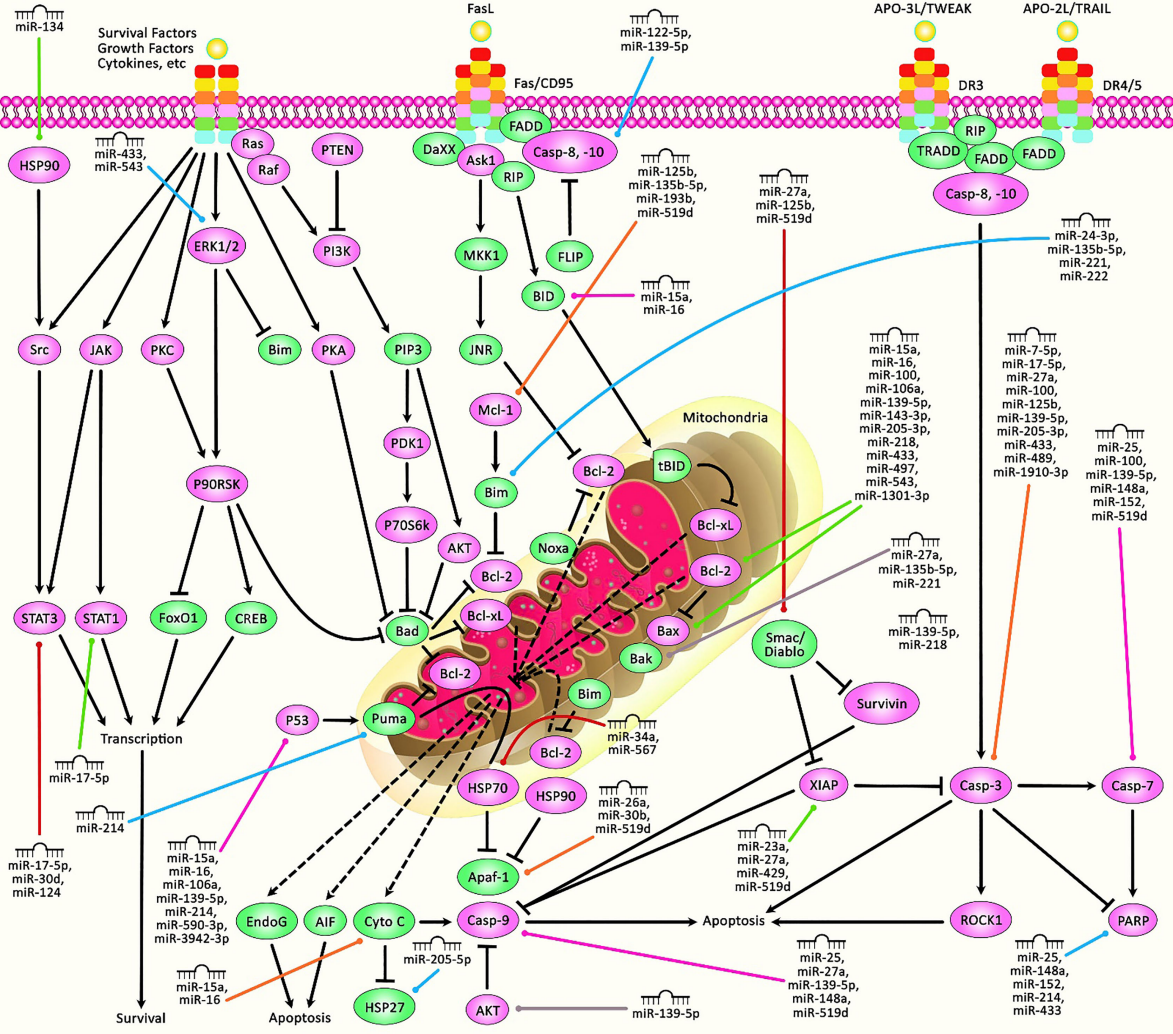
A schematic diagram of the role of miRNAs in triggering the mitochondrial cascade of apoptosis in human breast cancer. Apoptosis pathway could be activated *via* both extrinsic and intrinsic cascades. The intrinsic pathway is generally occurring through the release of cytochrome c from the mitochondria and modulates mitochondrial outer membrane permeabilization *via* Bcl-2 family proteins. The activation of extrinsic cascade could be triggered *via* ligand binding to death receptor, including DR3, DR4, DR5, Fas, and TNFαR. Following that, caspase proteins have a significant part in cleaving target proteins as well as nuclear lamins to elevate DNA degradation, leading to apoptotic cells undergoing phagocytosis. Furthermore, P53, *via* triggering the upregulation of various proteins containing Bid, Bax, CD95, Puma, and TRAIL-R2, could get effectively involved in activating intrinsic and extrinsic apoptosis cascades. Therefore, any alterations or abnormalities occurring during apoptotic pathways could considerably contribute to the progression of human diseases, including cancer. Previous studies have authenticated that several miRNAs could have a crucial role in regulating the apoptosis pathway in breast cancer. All the information regarding the role of these miRNAs involved in the modulation of breast tumors can be seen in **Table 1**.

Another study has demonstrated the impact of miR-23a on the suppression of apoptosis in breast cancer cells. Notably, this impact has been exerted in an independent manner from its inhibitory role on the X-linked inhibitor of apoptosis protein, the most potent anti-apoptotic member of the inhibitor-of-apoptosis proteins (21). Notably, the role of miR-23a on the enhancement of invasiveness of breast cancer cells has been verified in xenograft models (22). Several other upregulated miRNAs in breast cancer, such as miR-27a, miR-32, miR-205-3p, miR-221/222, and miR-1271, as well as downregulated miRNAs in breast cancer, such as miR-17-5p, miR-134, miR-139-5p, miR-200b, miR-214, miR-218, miR-543, miR-1301-3p, and miR-4458, have been found to regulate apoptosis in breast cancer cells. **Table 1** shows the regulation of apoptosis by miRNAs in breast cancer. **Figure 1** demonstrates that the aberrant expression of various miRNAs could contribute in adversely modulating the mitochondrial pathway of apoptosis which is involved in triggering human breast cancer.

**Table 1:** Regulation of apoptosis by miRNAs in breast cancer.

microRNA	Expression pattern	Samples	Cell lines	Target/pathway	Function	Reference
miR-7-5p	–	Nude mice, BC tissues	BT549, MDA-MB-231, MDA-MB-468, MCF-7, SK-BR-3, T47D, HBL100, MCF-10A	REGγ, p21, p27, Caspase-3	miR-7-5p, by targeting REGγ, could suppress cell proliferation and induces apoptosis of BC cells	(16)
miR-15a, miR-16	–	miRTarBase	MCF-7, MDAMB-231	BMI1, Bax, Bcl-2, BID, PARP, Caspase-3/9, Cyt-c, p21, p53	miR-15a and miR-16, by suppressing oncogene BMI1, could induce mitochondrial-dependent apoptosis in BC cells	(19)
miR-17-5p	Down	–	MCF-7, MDA-MB-231, MCF-7/tamoxifen, MDA-MB-231/paclitaxel	STAT1/3/5, p21/27/57/51/53, Bax, PARP, Caspase-3	miR-17-5p, by targeting STAT3 through inhibiting the STAT3/p53 pathway, could induce apoptosis in BC cell	(20)
miR-23a	–	Nude mice	MCF-7, T47D, SKBR3, BT549, MDA-MB-231, MDA-MB-435S, MCF-10A	XIAP, LC3-II/I, p62	miR-23a could promote survival and migration through modulating XIAP-mediated autophagy in BC cells. It can suppress apoptosis in breast cancer cells	(22)
miR-27a	Up	40 pairs of BC and ANTs	MCF-10A, T-47D, MDA-MB-231, BT-20, MCF-7	Bak, XIAP, Caspase-3/9, SMAC/DIABLO	miR-27a, *via* BAK-SMAC/DIABLO-XIAP axis, could regulate the sensitivity of BC cells to cisplatin treatment. This miRNA suppresses the apoptosis of breast cancer cells through regulation of the BAK-SMAC/DIABLO-XIAP axis	(23)
miR-32	Up	27 pairs of BC and ANTs	MCF-10A, MCF-7, MDA-MB-231	FBXW7	miR-32, by targeting FBXW7, could promote cell proliferation and suppress apoptosis in BC cells	(24)
miR-34a	–	Nude mice/human; 222 BC tissues and ANTs	MCF-10A,184A1, SKBR3, T47D, BT474, MCF-7, BT-483, BT-20, BT549, MDA-MB-468, MDA-MB-231	circGFRA1, GFRA1	circGFRA1, through sponging miR-34a, could regulate GFRA1 expression to exert regulatory functions in triple-negative BC. miR-34a increases the apoptosis of BC cells	(25)
miR-100	–	Nude mice	MCF-7, T47D, HCC1954, SK-BR-3, MDA-MB-453,	MTMR3, p27, Bcl-2, Bax, Cyclin-B, CDK1, Caspase-3/7	miR-100 is involved in regulating the apoptosis of BC cell	(26)
miR-106a	–	40 pairs of BC and ANTs	MDA-MB-231, MCF-7	P53, Bax, RUNX3, Bcl-2, ABCG2	miR-106a, by upregulating Bcl-2, ABCG2, and p53 and downregulating Bax and RUNX3, could promote BC cell proliferation and invasion and inhibit their apoptosis	(27)
miR-125b	–	–	MCF-7, MCF-7/DR, MCF-10A, T-47D, MDA-MB-435	Mcl-1, Caspase-3, PARP, smac/DIABLO, Cyt C	miR-125b and its synergistic effect on doxorubicin-inducing cell death, through the downregulation of Mcl-1 expression, resulting in mitochondria damage, and caspase-3 activation, could promote cell apoptosis in BC	(28)
miR-134	Down	77 pairs of BC and ANTs	Hs578T, Hs578Ts(i)8	STAT5B, Hsp90, Bcl-2	In extracellular vesicles, miR-134 could increase drug sensitivity in triple-negative BC and enhance their apoptosis	(29)
miR-139-5p	Down	GEO database	CBP60419, CBP60397, CBP60380, CBP60402, CBP60374	COL11A1, Caspase-3, Bax, Bcl-2	Overexpression of miR-139-5p, by inhibiting the COL11A1, could inhibit the proliferation and promote the apoptosis of BC cells	(30)
miR-139-5p	–	–	MCF-7, MCF-7/Doc	Notch1, Caspase-3/7/8/9, MMP2/7/9, Survivin, Akt, p53	miR-139-5p, by targeting Notch1, could inhibit the biological function of BC cells and mediate chemosensitivity to docetaxel	(31)
miR-143-3p	–	145 BC samples	MCF-10A, MDA-MB-435,	MYBL2, Bax, Bcl-2, Cyclin-B1, p21	miR-143-3p, by targeting MYBL2, could inhibit the proliferation and induce the apoptosis of BC cells	(32)
miR-148a, miR-152	–	36 pairs of ER^+^ BC with or without tamoxifen treatment, GEO datasets	MCF-7	ALCAM, PARP, Caspase-7/9	miR-148a and miR-152, by downregulating ALCAM, could reduce tamoxifen resistance in ER^+^ BC	(33)
miR-152	–	41 pairs of BC and ANTs	MCF-7, MDA-MB-231, MCF-10A	KIF4A, ZEB1	Circular RNA KIF4A, *via* miR-152/ZEB1 axis, could promote cell migration and invasion and inhibit apoptosis in BC	(34)
miR-193b	–	–	MCF-7, MCF-7/Dox	MCL-1	miR-193b, by downregulating MCL-1, could modulate the resistance of BC cells to doxorubicin and increase their apoptosis	(35)
miR-199a-3p	–	–	MDA-MB-231, MDA-MB-231/DDP	TFAM	miR-199a-3p, by downregulating TFAM, could enhance BC cell sensitivity to cisplatin	(36)
miR-200b	Down	278 pairs of BC and ANTs	MDA-MB-231, SK-BR-3, MCF-7, MDA-MB-468, HBL-100	Sp1	miR-200b, by targeting Sp1, could induce apoptosis and inhibit cell proliferation in BC	(37)
miR-205-3p	Up	58 pairs of BC and ANTs	MCF‐7	Ezrin, LaminA/C, Caspase-3, Bcl-2, Bax	Overexpression of miR‐205‐3p could promote proliferation and invasion and reduce the apoptosis of BC cells and reduce the survival time of patients	(38)
miR-214	Down	31 pairs of BC and ANTs	MCF-7, MDA-MB-157, MDA-MB-468, MCF-7/Dox, MDA-MB-157/Dox	RFWD2, p53, PUMA, p21, PARP	miR-214, by targeting the RFWD2-p53 axis, could promote apoptosis and sensitize BC cells to doxorubicin	(39)
miR-214, miR-218	Down	49 pairs of BC and ANTs	MCF-7	–	Overexpression of miR-214 or miR-218 could suppress cell proliferation and migration, disturb the cell cycle, and induce cell apoptosis in BC	(40)
miR-218	–	Nude mice	MCF-7, Cal51, MCF-7/A02, CALDOX	Survivin, Bax, Bcl-2	miR-218, *via* targeting surviving, could regulate resistance to chemotherapeutics in BC	(41)
miR-221	Up	35 pairs of BC and ANTs	MDA-MB-231, BT-20, MDAMB-435, T-47D, MCF-10A	BIM-Bax/Bak	Anti-miR- 221, by targeting the Bim-Bax/Bak axis, could promote the cisplatin-inducing apoptosis in BC	(42)
miR-221/222	Up	Nude mice/human; 48 pairs of BC and ANTs	MCF-7, MDA-MB-231, MDA-MB-453, SKBR3, MCF-10A	GAS5	miR-221/222, *via* lncRNA GAS5 in BC, could promote tumor growth and suppress apoptosis	(43)
miR-429	–	–	MDA-MB-231, MDA-MB-468	XIAP	miR-429, by targeting XIAP, could mediate δ-tocotrienol-induced apoptosis in triple-negative BC cells	(44)
miR-433	Down	Mice	4T1, MCF-7, 293T	MAPK/ERK, Rap1a, MMP-9, Caspase-3, Bax, Bcl-2, PARP1, p38	miR-433 *via* the MAPK signaling pathway, by targeting Rap1a, could inhibit BC cell growth	(45)
miR-451	Down	TCGA database	MCF-7, SKBR3, MCF-7/PR, SKBR3/PR	YWHAZ, β-catenin, c-Myc, Cyclin-D1	miR-451, by regulating YWHAZ in SKBR3/PR, drug resistant, could induce tumor suppression in BC	(46)
miR-497	Down	Nude mice and human; 45 pairs of BC and ANTs	T-74D, MCF-7, MDA-MB-453, MDA-MB-468, MDA-MB-435, MCF-10A	Bcl-2, Bax, α-SMA, E-cadherin, Vimentin, N-cadherin, Slug	miR-497, by targeting slug, could inhibit EMT transition in BC	(47)
miR-519d	–	Nude mice with or without Cisplatin	T-47D, MCF-7, SKBR3, MCF-10A	MCL-1, Caspase-3/7/9, Apaf-1, Smac/DIABLO, Cyt C, Xiap	miR-519d, by downregulating MCL-1, could impede cisplatin resistance in BC stem cells	(48)
miR-543	Down	–	MDA-MB-231, MCF-7	MAPK/ERK, Cyclin-D1, Bcl-2, Bax, RSK2, MSK1, ERK2	miR-543, by targeting ERK/MAPK, could suppress BC cell proliferation, block cell cycle, and induce cell apoptosis	(49)
miR-590-3p	–	–	MCF-7, MDA-MB231	Sirtuin-1, p53, p21, Bax	miR-590-3p, by targeting sirtuin‐1 and deacetylation of p53, could suppress cell survival and trigger BC cell apoptosis	(50)
miR-1271	Up	36 pairs of BC and ANTs	MCF-7, MDAMB-231, MDA-MB-468, MDA-MB-453, MCF-10A	circ-ABCB10	circ-ABCB10 could promote BC proliferation and progression *via* sponging miR-1271	(51)
miR-1301-3p	Down	60 pairs of BC and ANTs	MCF-7, T47D, MDA-MB-231, MDA-MB-468, MCF-10A	ICT1, CDK4, p21, Cyclin-D1, Bcl-2, Bax, Bad	miR-1301-3p, by targeting ICT1, could inhibit BC cell proliferation by regulating cell cycle progression and apoptosis	(52)
miR-3942-3p	–	GEO database, 15 pairs of tissues with or without TCDD (2, 3, 7, 8-tetrachlorodibenzo-p-dioxin) treatment	MCF-7, MCF-7/TCDD	Hsa_circ_0001098 (BARD1), γ-H2AX, p53	Overexpression of circular RNA BARD1 with TCDD treatment could promote cell apoptosis *via* miR-3942 in BC cells	(53)
miR-4301	–	NCBI database, 30 pairs of BC and ANTs	MDA-MB-231, MCF-7, SKBR3, MCF-10A	DRD2	miR-4301, by negatively regulating DRD2 expression, could induce cell apoptosis in human BC cells	(54)
miR-4458	Down	60 pairs of fresh TNBC and ANTs	MCF-10A, BT549, MDA-MB-436	SOCS1	miR‐4458, by targeting SOCS1, could suppress cell proliferation and promote cell apoptosis in triple‐negative BC	(55)

ANTs, adjacent normal tissues.

## Regulation of Autophagy by miRNAs in Breast Cancer

**Figure 2:**
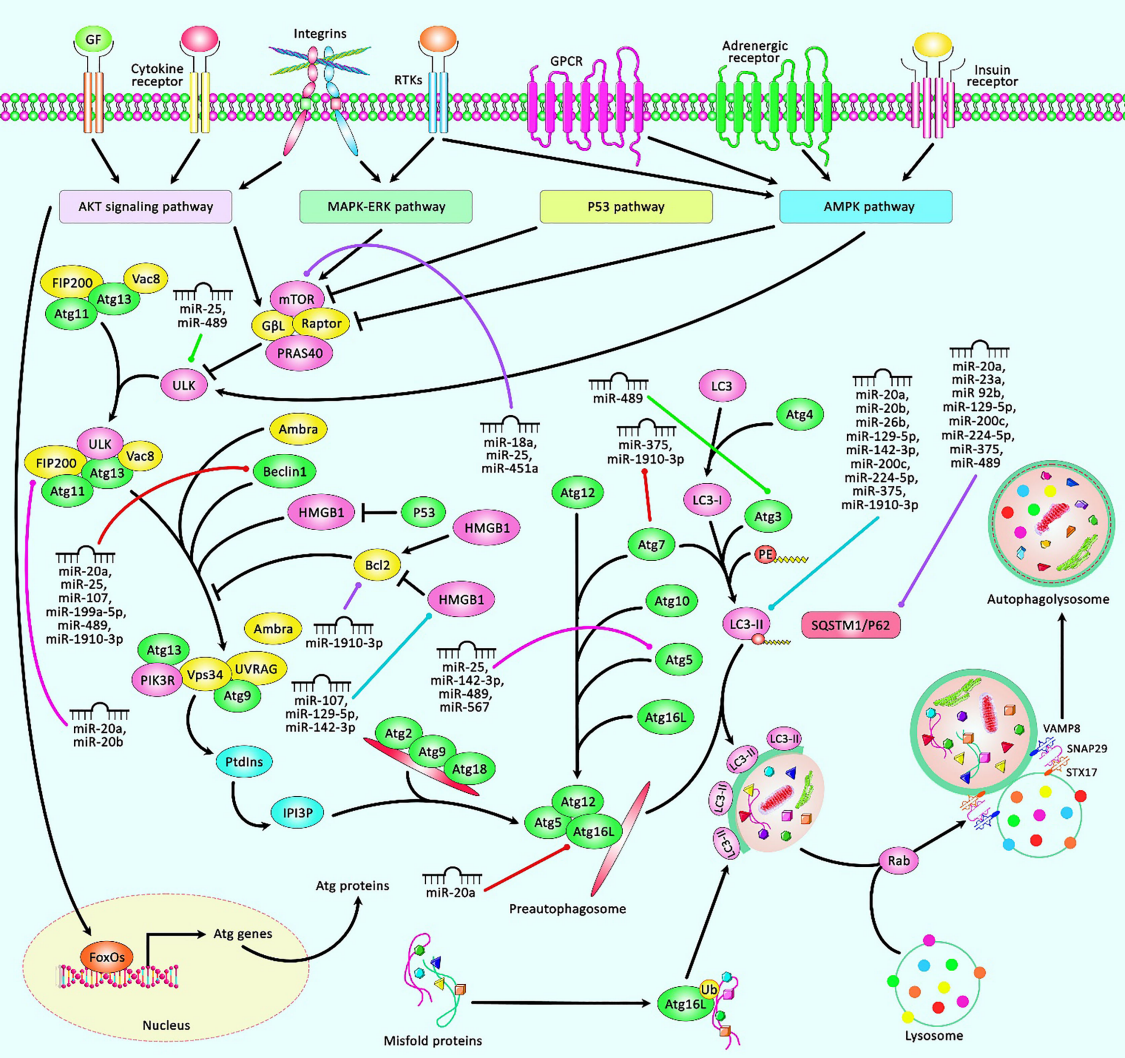
A schematic representation of the role of several miRNAs in regulating the autophagy cascade in human breast cancer. The autophagy pathway is comprised of multiple sequential steps containing sequestration, transport to lysosomes, and degradation. The expression of Atgs could be triggered *via* AKT, MAKP-ERK, P53, and AMPK pathways. Autophagy is a fundamental substantial biological cascade by removing damaged organelles, but dysregulation of autophagy could contribute to several diseases, including cancers. Accumulating evidence has illustrated that various miRNAs could have a remarkable part in modulating the apoptosis cascade in breast tumors. All the information regarding the role of these miRNAs contributing to the regulation of breast cancer can be seen in **Table 2**.

An autophagy mechanism is initiated by the establishment of autophagosomes that seizure degraded apparatuses and then fuse with lysosomes to induce recycling processes. Autophagy has dual impacts in tumor inhibition and promotion in several types of malignancies. Moreover, autophagy influences cancer stem cell properties through participating in the maintenance of stemness, regulation of tumor recurrence, and induction of resistance to anticancer drugs (56). Autophagy is another subject of regulation by miRNAs in breast cancer cells. miR-20a is among the upregulated miRNAs in breast cancer, particularly in triple-negative breast cancer cells. The expression of miR-20a has been negatively correlated with the activity of the autophagy/lysosome pathway. miR-20a suppresses the basal and nutrient starvation-associated autophagic flux and activity of lysosomal-associated proteolysis. Moreover, this miRNA enhances the intracellular ROS levels and DNA damage response through modulating numerous important regulators of autophagy; among them are BECN1, ATG16L1, and SQSTM1. The expression of miR-20a has been negatively correlated with the expressions of these target genes in breast cancer tissues. Notably, triple-negative cancers have exhibited a particular downregulation of BECN1, ATG16L1, and SQSTM1 genes. The upregulation of miR-20a has also been associated with a higher occurrence of copy number variations and genetic mutations in breast cancer samples. The effects of miR-20a on the enhancement of tumor evolution and growth have also been confirmed in a xenograft model of breast cancer (57). Another study has shown the regulatory effects of miR-20a and miR-20b on the expression of RB1CC1/FIP200. Both miRNAs could decrease the expression of RB1CC1/FIP200 transcripts and proteins. The upregulation of these miRNAs has reduced basal and rapamycin-associated autophagy. Therefore, miR-20a and miR-20b can regulate autophagy through influencing the expression of RB1CC1/FIP200 (58). A high-throughput miRNA sequencing experiment has reported miR-25 as the most important target of isoliquiritigenin (ISL) in inducing autophagy flux. Moreover, mechanistical studies have shown that miR-25 silencing results in cell autophagy through enhancing the expression of ULK1, an early regulator of autophagy initiation. miR-25 upregulation blocks ISL-associated autophagy. ISL has been found to sensitize cancer cells to chemotherapeutic agents as demonstrated by the enhancement in LC3-II levels, decrease in ABCG2 levels, downregulation of miR-25, and activation of ULK1 (59). The inhibitory roles of miR-26b, miR-129-5p, and miR-200c on autophagy are exerted through the modulation of DRAM1 (60), HMGB1 (61), and UBQLN1 (62) expressions, respectively. Notably, miR-129-5p and miR-200c could attenuate irradiation-induced autophagy and decrease the radioresistance of breast cancer cells through this route (61) (62). **Table 2** shows the regulation of autophagy by miRNAs in breast cancer. **Figure 2** presents the role of several miRNAs in breast cancer cells *via* regulating the autophagy pathway.

**Table 2:** Regulation of autophagy by miRNAs in breast cancer.

microRNA	Expression pattern	Samples	Cell lines	Target/pathway	Function	Reference
miR-20a	Up	TCGA database, nude mice and human; 83 pairs of BC and ANTs	MDA-MB-231 and MCF7	LC-3 I/II, BECN1, SQSTM1, ATG16L1, OPTN, γH2AX	miR-20a-mediated loss of autophagy could be involved in breast tumorigenesis	(57)
miR-20a, miR-20b	Down	19 pairs of breast cancer tissue and ANTs	MCF7, MDA-MB-231	FIP200, LC-3 I/II, p62	miR-20a and 20b, downregulated by suppressing RB1CC1/FIP200, could modulate autophagy in breast cancer	(58)
miR-25	–	Nude mice	MCF-7, MCF-7/ADR	ABCG2, ULK1, LC-3 I/II, BECN1, Atg5, Bcl-2, Caspase-6/7/9, PARP, Bax, mTOR	miR-25 could regulate chemoresistance-associated autophagy in BC cells	(59)
miR-26b	Down	3 pairs of BC and ANTs	MCF7	DRAM1, LC-3 I/II	miR−26b, by targeting DRAM1, could suppress autophagy in breast cancer cells	(60)
miR-129-5p	–	–	MCF-7, MDA-MB-231, BT474, BT549, MCF-10	HMGB1, LC-3 I/II, p62, Caspase-3, PARP	miR-129-5p, by targeting HMGB1, could attenuate irradiation-induced autophagy and decrease the radioresistance of BC cells	(61)
miR-200c	–	35 pairs of BC and ANTs	MDA-MB-231, BT549, MCF-10A, BT474, MCF-7	UBQLN1, LC-3 I/II, p62, Caspase-3, PARP	miR-200c cells, by targeting UBQLN1, could inhibit autophagy and enhance radiosensitivity in breast cancer	(62)
miR-375	–	–	MCF-7	HR, PR, Her2, EGFR, C-Abl, Crkl, ATG7, p62, LC31/2	miR-375-autophagy axis could suppress the growth of fulvestrant-resistant breast cancer cells by the combined inhibition of EGFR and c-ABL	(63)
miR-224-5p	Up	30 pairs of BC and ANTs	MDA-MB-231, MCF-7	Smad4, SQSTM1, LC-3 I/II	miR-224-5p, *via* targeting Smad4, could inhibit autophagy in breast cancer cells	(64)
miR-451a	–	–	MCF-7, LCC2	14-3-3ζ, ERα, mTOR, AKT, LC-3 I/II	Over-expression of miR-451a, by regulating 14-3-3ζ, estrogen receptor α, and autophagy, could enhance the sensitivity of breast cancer cells to tamoxifen	(65)
miR-142-3p	–	Nude mice	MCF-7, MCF-7/DOX	HMGB1, ATG5, LC-3 I/II	miR-142-3p by targeting HMGB1 could enhance chemosensitivity of breast cancer cells and inhibits autophagy.	(66)
miR-1910-3p	Up	Nude mice and human; 55 pairs of BC and ANTs	MCF-7, MDA-MB-231, MCF-10A	MTMR3, NF-κB, PCNA, Bcl2, p65, IκBα, LC3B, ATG7, BECN1, PARP, Caspase-3, E-cadherin, N-cadeherin, Vimentin, Slug, Twist	Exosomal miR-1910-3p, by targeting MTMR3 and activating the NF-κB signaling pathway, could promote the proliferation, metastasis, and autophagy of breast cancer cells	(67)
miR-489	–	GEO database, nude mice and human BC tissue	MDA-MB-231, HCC1954, T47D	LC3B-I, LC3B-II, p62, ATG5/3, Beclin1, ULK1, LAMTM4B, Caspase-3	miR-489 could regulate autophagy, cell viability, and chemoresistance in breast cancer	(68)
miR-129-5p	–	Oncomine databases	MCF-7	HMGB1, p62, LC3B-I, LC3B-II	Upregulation of miR-129-5p, through inhibiting HMGB1-mediated cell autophagy, could increase the sensitivity to Taxol in breast cancer MCF-7 cells	(69)
miR-18a	–	–	MDA-MB-231, MDA-MB-231/PTX, MCF-10A	p70S6, mTOR, LC3 I, LC3 II	miR-18a upregulation, *via* inhibiting mTOR signaling pathway, could enhance autophagy in triple-negative cancer cells	(70)
miRNA‐107 family	–	Nude mice and human; 62 pairs of BC and ANTs	MDA‐MB‐231, MDA‐MB‐453, MCF‐10A, MCF‐7	HMGB1, p62, Beclin1	miR‐107 family, by targeting HMGB1, could inhibit the autophagy, proliferation, and migration of breast cancer cells	(71)
miR−92b	–	30 pairs of BC and ANTs	MCF-7, MDA-MB-453	EZH2, LC3 I, LC3 II, SQSTM1	miR−92b, by targeting EZH2, could promote autophagy and suppress viability and invasion in breast cancer	(72)
miR-199a-5p	–	–	MCF7, MDA-MB-231	LC3 I, LC3 II, DRAM1, Beclin1	miR-199a-5p could be involved in radiation-induced autophagy	(73)

ANTs, adjacent normal tissues.

## Regulation of EMT by miRNAs in Breast Cancer

**Figure 3:**
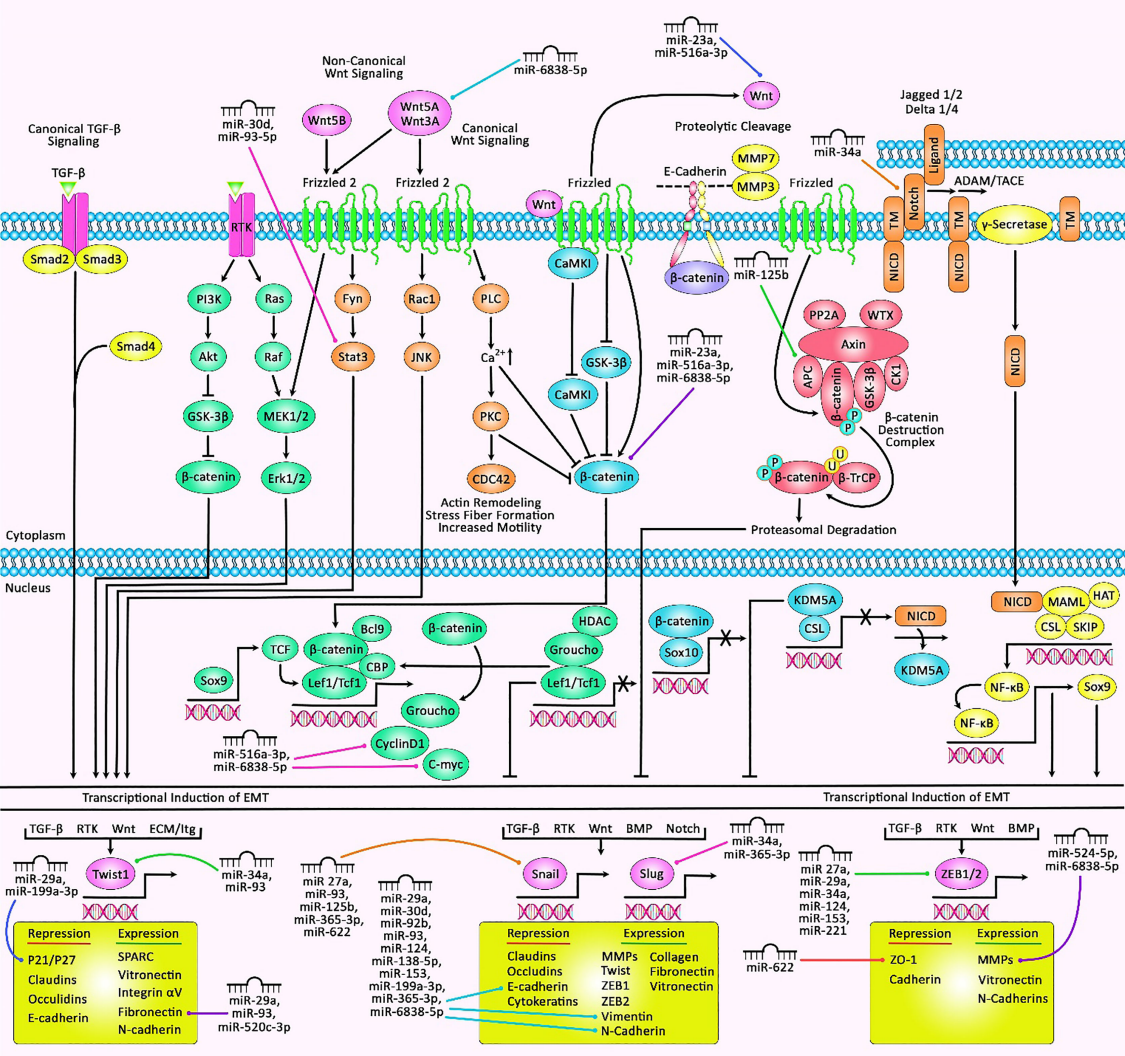
A schematic illustration of the epithelial–mesenchymal transition (EMT)‐associated miRNAs and their roles in human breast cancer. EMT is a process that can be induced *via* a variety of growth factors and cytokines in cancer cells. These elements may be secreted through the cancer cells themselves or *via* the stromal cells in the tumor microenvironment. These soluble ligands can interact with their cognate receptors, such as TGF-β receptors and RTKs, resulting in the activation of several oncogenic pathways (TGF-β, Wnt/β-catenin, integrins, Notch, *etc.*) which have a significant role in inducing the EMT cascade. Thereby, the activation of EMT can be triggered through the overexpression of selected zinc finger, including ZEB1/2, snail, slug, or basic helix–loop–helix containing TWIST1 transcription factors (95, 96). Recent studies have detected he regulatory role of multiple miRNAs in EMT and breast cancer cells. All the information regarding the influence of these miRNAs in EMT and the control that they exert in major signaling cascades in breast cancer can be seen in **Table 3**.

EMT is a complicated developmental program that permits carcinoma cells to change the epithelial characteristics to mesenchymal features. This alteration permits them to obtain mobility and migration ability. EMT is involved in numerous stages of the metastatic program, from dedifferentiation to aggressiveness (74). TGF-β1-induced EMT has been shown to participate in the metastasis of breast cancer cells. This process is regulated by a number of miRNAs—for instance, miR-23a as an upregulated miRNA in breast cancer cells, particularly in metastatic samples, has been shown to be induced by TGF-β1. The TGF-β1-associated regulation of miR-23a is mediated by direct binding of Smads with the RNA Smad-binding element in miR-23a. The suppression of miR-23a expression has inhibited TGF-β1-associated EMT and attenuated the migration, invasiveness, and metastatic ability of breast cancer cells. miR-23a can directly inhibit the expression of CDH1, a key modulator of EMT. The miR-23a-mediated suppression of CDH1 has been found to activate Wnt/β-catenin signaling. Taken together, miR-23a enhances TGF-β1-associated breast cancer metastasis through influencing the expression of CDH1 and inducing Wnt/β-catenin cascade (75). miR−27a is another upregulated miRNA in breast cancer samples and cell lines. The upregulation of miR−27a has increased the migratory potential of breast cancer cells through induction of EMT. FBXW7 has been identified as a downstream target of miR−27a. The over-expression of FBXW7 in breast cancer cells could inhibit EMT and the migratory aptitude of these cells. Therefore, miR−27a can regulate the metastatic potential of breast cancer through the suppression of FBXW7 (76). miR-29a has also been found to be upregulated in breast cancer samples in correlation with distant metastasis and poor clinical outcome of patients. miR-29a silencing has suppressed the proliferation and migration of breast cancer cells. Ten eleven translocation 1 (TET1) has been identified as a target of miR-29a. The upregulation of TET1 has attenuated the proliferation and migration of breast cancer cells. The miR-29a-mediated downregulation of TET1 enhances EMT (77). Several upregulated miRNAs in breast cancer, such as miR-93, miR-125b, miR-199a-3p, and miR-221, as well as downregulated miRNAs, such as miR-34a, miR-92b, miR-124, miR-138-5p, miR-153, miR-516a-3p, and miR-524-5p, affect the EMT process. **Table 3** shows the regulation of EMT by miRNAs in breast cancer. **Figure 3** depicts the role of various miRNAs in the modulation of EMT *via* targeting receptors that convey signals from EMT inducers or multiple EMT components.

**Table 3:** Regulation of epithelial–mesenchymal transition (EMT) by miRNAs in breast cancer.

microRNA	Expression pattern	Samples	Cell lines	Target/pathway	Function	Reference
miR-23a	Up	30 pairs of BC and ANTs, nude mice	MCF-7, MDA-MB-468, T47D, BT-549, MDA-MB-231	CDH1, Wnt/β-catenin, E-cadherin	miR-23a, by targeting CDH1 and activating Wnt/β-catenin signaling, could promote TGF-β1-induced tumor metastasis in breast cancer	(75)
miR−27a	Up	20 pairs of BC and ANTs	MDA-MB-231, SKBR3, MCF-12A	FBXW7, ZEB1, Snail, Vimentin, E-Cadherin, N-Cadherin	miR−27a, by inducing EMT in a FBXW7−dependent manner, could promote human breast cancer cell migration	(76)
miR-29a	–	Nude mice and human; 60 pairs of BC and ANTs	MDA-231, MDA-453, MCF-7, MCF-10	TET1, CyclinD1, p21, E-Cadherin, N-Cadherin, Fibronectin, Vimentin, ZEB1, ZEB2	miR-29a, by targeting ten eleven translocation 1, could promote cell proliferation and EMT in breast cancer	(77)
miR-30d	–	–	BT474, MDA-MB-231, HCC197, MDA-MB-468	KLF11, STAT3, Bcl-2, Bax, Vimentin, N-cadherin, E-cadherin	miR-30d, by targeting KLF11 and activating the STAT3 pathway, could mediate breast cancer invasion, migration, and EMT	(78)
miR-34a	Down	48 pairs of BC and ANTs	MCF-7, T-47D, BT-549, MDA-MB-231, MDA-MB-435	SLUG, ZEB1/2, NOTCH1, TWIST1	miR-34a could inhibit BC cell migration and invasion *via* targeting EMT-inducing transcription factors	(79)
miR-92b	Down	51 pairs of BC and ANTs	MCF-10A, BT549, MDAMB-231	Gabra3, Vimentin, N-cadherin, E-cadherin	miR-92b, by targeting Gabra3, could inhibit EMT	(80)
miR-93	Up	16 pairs of BC and ANTs	MCF-7, MCF-7/ADR	Twist, Snail, fibronectin, Vimentin, N-cadherin, E-cadherin	miR-93 could induce EMT and drug resistance of BC cells by targeting PTEN	(81)
miR-93-5p	–	–	MCF-7, MDA-MB-231, T47D	MKL-1, STAT3, Vimentin, N-cadherin, E-cadherin	miR-93-5p, by targeting MKL-1 and STAT3, could inhibit the EMT of breast cancer cells	(82)
miR-124	Down	30 pairs of BC and ANTs	MDA-MB-453, MDA-MB-231, BT-549	Vimentin, N-cadherin, E-cadherin, ZEB2	miR-124, by regulating EMT based on ZEB2 target, could inhibit invasion and metastasis in triple-negative breast cancer	(83)
miR-125b	Up	20 pairs of BC and ANTs	MDA-MB-231, MCF-10A, MCF-7, MDAMB-468	Vimentin, E-cadherin, snail, APC, β-catenin, cyclin D	miR-125b, *via* the Wnt/β-catenin pathway and EMT, could regulate the proliferation and metastasis of triple-negative breast cancer cells	(84)
miR-138-5p	Down	TCGA dataset, 20 pairs of BC and ANTs	MDA-MB-231, MDA-MB-468, T47D, ZR-75-30	N-cadherin, E-cadherin, Vimentin, RHBDD1	miR−138−5p, by targeting RHBDD1, could inhibit cell migration, invasion, and EMT in breast cancer	(85)
miR-153	Down	60 pairs of TNBC and ANTs	SKBR3, BT-549, MDA-MB-231, MCF-10A	ZEB2, E-cadherin, N-cadherin, Vimentin	miR-153, through targeting ZEB2-associated EMT, could inhibit the progression of triple-negative breast cancer	(86)
miR-199a-3p	Up	–	HCC1806, HCC1937, MDA-MB-231, HMEC-184	GPER, p21, CDK2, Cyclin E1, Vimentin, N-cadherin, E-cadherin, VEGFA, Ang II, CD151	Through CD151/miR-199a-3p bio-axis, the activation of GPER could inhibit cell proliferation, invasion, and EMT of triple-negative breast cancer	(87)
miR-221	Up	TCGA database	BT549, HCC1806, MDA-MB-231, T47D, MDA-MB-468, MCF7, MDA-MB-361, SKBR3	ZEB1, MAPK, uPAR, Vimentin, HER2, ER, PR	A combined treatment (MEK1 inhibitor + irradiation) could decrease the migratory potential of BC cells by reducing miR-221. This miRNA induces EMT in these cells	(88)
miR-365-3p	–	93 pairs breast cancer tissue and ANTs	MCF-7, MDA-MB-231, MCF-10A	FOXK1, Vimentin, N-cadherin, E-cadherin, Slug, Snail	miR-365-3p, by regulating FOXK1, could promote cell growth and EMT indicates unfavorable prognosis in breast cancer	(89)
miR‐516a‐3p	Down	Nude mice and human; 60 pairs breast cancer tissue and ANTs	MDA‐ MB‐231, MCF‐7, HEK293T	Pygo2, Wnt, E-cadherin, Vimentin, c‐Myc, cyclinD1, β‐catenin	miR‐516a‐3p, by blocking the Pygo2/Wnt signaling pathway, could inhibit breast cancer cell growth and EMT	(90)
miR-520c-3p	–	–	MCF-7 and T47D, 293T	IL-8, E-cadherin, Vimentin, fibronectin	miR-520c-3p, by targeting IL-8, could negatively regulate EMT to suppress the invasion and migration of breast cancer	(91)
miR-524-5p	Down	20 pairs breast cancer tissue and ANTs	SK-BR-3, MDA-MB-453	FSTL1, MMP2, MMP9, E-cadherin, N-cadherin	miR-524-5p, through targeting FSTL1, could suppress migration, invasion, and EMT	(92)
miR-622	–	GEO and TCGA dataset	MDA-MB-231, MCF7	RNF8, E-cadherin, ZO-1, Snail	The miR-622 induces EMT through modulation of the expression of RNF8	(93)
miR‐6838‐5p	–	–	CC1937, HCC70, MDA‐MB‐231, MDA‐MB‐436, MDA‐MB‐468	WNT3A, MMP2/9, E-cadherin, N-cadherin, Vimentin, β‐catenin, c‐myc, Cyclin-D1	miR‐6838‐5p, by targeting WNT3A to inhibit the Wnt pathway, could suppress cell metastasis and the EMT process in triple‐negative breast cancer	(94)

ANTs, adjacent normal tissues.

## Regulation of Chemoresistance Breast Cancer Cells by miRNAs

Chemoresistance is a phenotype which is associated with several signaling pathways as well as cellular processes such as apoptosis, autophagy, and EMT. miRNAs have also been found to affect the resistance of breast cancer cells to important chemotherapeutic drugs—for instance, miR-7 has been shown to be downregulated in MCF-7 and adriamycin-resistant cells (MCF-7/ADR cells), particularly in MCF-7/ADR cells. The upregulation of miR-7 has enhanced sensitivity of MCF-7/ADR cells to ADR. The downregulation has led to the upregulation of EGFR and PI3K, while the upregulation of miR-7 has been associated with opposite effects. Moreover, the suppression of miR-7 has been associated with the enhancement of proliferation and inhibition of apoptosis. Therefore, miR-7 has been found to affect the resistance of breast cancer cells to ADR, and its upregulation can enhance the effects of ADR through the suppression of EGFR/PI3K signaling (97). miR-30c is another miRNA that is involved in intrinsic adriamycin resistance in p53-mutated breast cancer (98).

Moreover, another study in breast cancer has shown a correlation between high miR-7 levels and better pathological complete response to paclitaxel/carboplatin. Functionally, miR-7 has been shown to sensitize MCF-7 and MDA-MB-231 cells to the cytotoxic effects of paclitaxel and carboplatin through targeting MRP1 and BCL2. Taken together, miR-7 has been suggested as a predictive marker for the assessment of chemotherapy efficacy and therapeutic target for the enhancement of response of breast cancer patients to chemotherapy (99). The expression assays in an Src inhibitor saracatinib-resistant breast cancer cell line (SK-BR-3/SI) has shown the downregulation of miR-19b-3p in saracatinib-resistant cells compared with saracatinib-sensitive ones. The under-expression of miR-19b-3p not only has been associated with higher IC50 value of saracatinib but also has increased the migratory potential of breast cancer cells. Functionally, miR-19b-3p targets PIK3CA. Thus, the resistance to Src inhibitors might be due to the enhancement of the activity of PI3K/Akt pathway following miR-19b-3p downregulation (100). In addition, miR-34a could affect the sensitivity of breast cancer cells to sunitinib by regulating the Wnt/β-catenin signaling pathway (101).

miR-24-3p is another miRNA which can regulate the sensitivity of breast cancer cells to tamoxifen. The upregulation of miR-24-3p has been shown to increase tamoxifen-induced cytotoxicity in breast cancer cells, while its silencing has decreased these effects. Bim has been identified as a target of miR-24-3p in breast cancer. Further experiments have shown the upregulation of miR-24-3p and the downregulation of BIM expression in tamoxifen-resistant MCF7 cells compared with original cells. Moreover, the suppression of miR-24-3p has enhanced the sensitivity of MCF7/TAM cells to tamoxifen through the enhancement of cell apoptosis (102). Besides this, miR-148a and miR-152, by downregulating ALCAM, could reduce tamoxifen resistance in ER+ breast cancer cells (33). miR-375 is another miRNA that could inhibit cancer stem cell phenotype and tamoxifen resistance in human ER+ breast cancer cells through degrading HOXB3 (103). Meanwhile, tamoxifen has been shown to regulate the expressions of miR-29b-1 and miR-29a (104). **Table 4** shows the role of miRNAs in the regulation of response of breast cancer to therapeutic agents.

**Table 4:** Role of miRNAs in the regulation of response of breast cancer to therapeutic agents.

microRNA	Expression pattern	Samples	Cell lines	Target/pathway	Function	Reference
miR-7	–	–	MiR-7, MCF-7/ADR, MCF-10A	EGFR/PI3K	miR-7 over-expression could inhibit the EGFR/PI3K signaling pathway to raise their sensitivity to the chemotherapy drug adriamycin	(97)
miR-7	–	60 pairs of BC tissue with or without paclitaxel plus carboplatin	MCF-7, MCF-7-PR, MDA-MB-231, HEK293	MRP1, BCL2	miR-7, by suppressing MRP1 and BCL2, could reverse breast cancer chemoresistance	(99)
miR-19b-3p	–	–	MDA-MB-231, SK-BR-3, BT-474, MCF-7	MDR-1,Src, PI3K/Akt	miR-19b-3p, by regulating the PI3K/Akt pathway, could inhibit breast cancer cell proliferation and reverse saracatinib resistance	(100)
miR‐24‐3p	Up	20 pairs of ER+ BC and ANTs	MCF7, MCF7/TAM, T47D	Bim, ER, PR, pS2, Caspase3, PARP	miR‐24‐3p overexpression, *via* direct repression of Bim expression, could promote the development of tamoxifen resistance in breast cancer cells	(102)
miR-26a, miR-30b	–	–	BT474 wt, BT474r, HCC1954, MDA-MB-231	APAF1, CCNE2, CASP3	The mentioned microRNAs could be involved in trastuzumab resistance	(105)
miR-30c	Up	TCGA database, nude mice and human BC tissue and ANTs	MCF-7, ZR-75-1, T-47D, MCF-10A, MDAMB-231	REV1, FANCF, FANCD2, RAD51, ATM, BRCA1, ERCC1, p53, p21	miR-30c could be involved in intrinsic adriamycin resistance in p53-mutated breast cancer	(98)
miR-34a	–	–	MCF-7	Wnt/β-catenin	miR-34a, by regulating the Wnt/β-catenin signaling pathway, could increase the sensitivity to sunitinib in breast cancer	(101)
miR-34a	–	–	MCF-7, MCF-10A, MDA-MB-231, BT-20, T47-D, PC3, DU-145, LNCaP, OVCAR, SK-OV-3, HeLa	HDAC1/7, HSP70, LC3-II/I	miR-34a, by targeting HDAC1 and HDAC7, could reduce therapy resistance in breast cancer	(106)
miR‐122‐5p	–	–	MCF‐7, MCF-7‐ADR	Bcl-2, CDK2/4/6, Caspase-8/9	Resveratrol could increase the sensitivity of BC *via* targeting the miR-122-5p/Bcl-2 axis. miR-122-5p enhances the chemosensitivity of BC cells	(107)
miR−124	Up	Nude mice and human BC tissue and ANTs	BT474, MCF7, SKBR3, MDA-MB-231	MCT1, LDHA	Restoration of MCT1 in miR-124-overexpressing cells could promote resistance to paclitaxel	(108)
miR-125b	–	–	MCF-7, MCF-7/PR, SKBR3, SKBR3/PR	Sema4C, Snail, Slug, Vimentin, E-cadherin	miR-125b, by targeting Sema4C, could regulate EMT in paclitaxel-resistant breast cancer cells	(109)
miR-129-3p	–	Nude mice	MDA-MB-231, MDA-MB-231/Doc, MCF-7	CP110	miR-129-3p, by CP110 inhibition, could promote docetaxel resistance of breast cancer cells	(109)
miR-137	–	Nude mice	MCF-7, MCF-7/ADR (adriamycin-resistant), HCC1937, MDA‐MB‐468	DUSP4, E-cadherin, Vimentin	miR-137, by targeting DUSP4 through inhibition of EMT, could alleviate doxorubicin resistance in breast cancer	(110)
miR-140-5p	–	Nude mice and human; 30 pairs of BC with or without paclitaxel	MCF-10A, MCF-7, MCF-7/PTX, MDA-MB-231, MDA-MB-231/PTX	E2F3	miR-140-5p, by upregulating E2F3, could improve the paclitaxel resistance of BC	(111)
miR-148a, miR-152	–	36 pairs of ER^+^ BC with or without tamoxifen, GEO datasets	MCF-7	ALCAM, PARP, Caspase-7/9	miR-148a and miR-152, by downregulating ALCAM, could reduce tamoxifen resistance in ER^+^ BC	(33)
miR-155-3p	Down	TCGA database, nude mice and human;10 pairs of BC tissue and ANTs	MCF-10A, MCF-7, MCF-7/PR, SKBR-3, MDA-MB-231	MYD88, Bcl-2, Bak-1, Bax, Caspase-3	miR-155-3p, by the negative regulation of MYD88, could act as a tumor suppressor and reverse paclitaxel resistance in human breast cancer	(83)
miR-200	–	–	MCF7/TAM, MCF-7, T47D	Vimentin, ZEB1/2, c-MYB	miR-200, by regulation of MYB, affects tamoxifen resistance in breast cancer cells	(112)
miR-200c	–	GEO database, nude mice	SKBr-3	Vimentin, E-cadherin, smad3, ZEB1	miR-200c, by targeting ZNF217 and ZEB1, could suppress TGF-b signaling and counteract trastuzumab resistance and metastasis in breast cancer	(113)
miR-222	Up	25 pairs of BC with or without doxorubicin	MCF-7, MCF-7-R	Bim, Caspase-9/3	miR-222, by regulation of miR-222/bim pathway, could promote drug resistance to doxorubicin in breast cancer	(114)
miR-326	–	35 pairs of BC and ANTs	MCF-7, MCF-7/VP (VP-16-resistant MCF-7)	MDR-1, MRP-1, BCRP,	miR-326 overexpression, by transfection of miR-326 mimic, could downregulate the expression of MRP-1 and also sensitize MCF-7/VP MDR cells to cytotoxic drugs in breast cancer	(115)
miR-375	–	–	MCF-7	HOXB3, TWIST, Cd133, Cd44, MTdH	miR-375, by degrading HOXB3, could inhibit cancer stem cell phenotype and tamoxifen resistance in human ER-positive breast cancer	(103)
miR-381	Down	46 pairs of BC tissue and ANTs	MCF-7, MCF-7/DDP MDA-MB-231, MDA-MB-231/DDP	MDR1	miR-381, by targeting MDR1, could overcome cisplatin resistance in breast cancer	(116)
miR-381	–	Nude mice and human;48 pairs of BC tissue and ANTs, TCGA database	MCF-7, MCF-7/CDDP, MDA-MB-231, MDA-MB-231/CDDP, MCF-10A	EZH2	EZH2 knockdown, through epigenetically silencing miR-381, could improve the cisplatin sensitivity of breast cancer cells	(117)
miR-423	Up	Nude mice and human; 40 pairs of BC tissues and ANTs	MCR-7, MCF-7/ADR	ZFP36, β-catenin	miR-423, *via* the Wnt/β-catenin signaling pathway, could inhibit the expression of ZFP36 in breast cancer cells. This miRNA induces chemoresistance	(118)
miR-489	–	Nude mice, BC tissue	MCR-7, MCF-7/ADM	Smad3	miR-489 downregulation or gain of Smad3 is a potential modulator of both chemoresistance and EMT-like properties in breast cancers. The expression of miR-489 was decreased in chemoresistance MCF-7/ADM cells compared with chemosensitive cells. Upregulation of miR-489 enhanced the chemosensitivity	(119)
miR-520h	–	–	MCF-7, MCF-7/Taxol	OTUD3-PTEN, p-AKT	miR-520h, by targeting the OTUD3-PTEN axis, could stimulate resistance to paclitaxel	(120)
miR-567	Down	GEO database, nude mice and human;60 pairs of BC tissue and ANTs	SKBR-3, BT474, SKBR-3-TR, BT474-TR	p62, LC3-I, LC3-II, ATG5, TSG101, HSP70	Exosome-transmitted miR-567 reverses trastuzumab resistance by inhibiting ATG5 in breast cancer	(121)
miR−873	–	–	MDA-MB-231, MDA-MB-231GEMr, BT549	ZEB1, E-cadherin, AXL, CTGF, CYR61	Loss of miR−873, *via* targeting ZEB1, could contribute to gemcitabine resistance in triple−negative breast cancer	(122)
miR-1246	Up	75 pairs of BC and ANTs	MCF-7, MDA-MB-231, MCF-10A, HMLE	CCNG2, tsg101, calnexin	miR-1246, by targeting CCNG2 in breast cancer, could promote cell proliferation, invasion, and drug resistance	(123)
miR-15a, miR-16	–	–	MCF-7, MDAMB-231	BMI1, RING1A, RING1B, EZH2, γ-H2AX, Ub-H2A, CHK2, ATM, RNF8, RNF168, MEL18, p53BP, BRCA1, p21, p53, CDK1, Cyclin-B1	These miRNAs enhance the sensitivity of breast cancer cells to DNA damage conferred by doxorubicin	(124)
miR-27b	–	GEO Datasets	MCF-7, MCF-7/TamS	HMGB3, E-cadherin, N-cadherin	miR-27b, by targeting HMGB3, could regulate tamoxifen sensitivity	(125)
miR-29b-1, miR-29a	–	–	MCF-7, LCC2, LCC9, LY2	ERα, DICER	Tamoxifen could regulate miR-29b-1 and miR-29a expression	(104)
miR-33a	–	–	SUM149, SUM159, KPL4 IBC, MDA-MB-231	ABCA1	miR-33a could decrease high-density lipoprotein-induced radiation sensitivity	(125)
miR-107	–	–	MCF-7, Taxol/miR-107	Bax, Bcl-2, Akt, TRIAP1	miR-107, by targeting TRIAP1, could regulate chemodrug sensitivity in mammary cancer cell	(126)
miR-107	Down	35 pairs of breast cancer tissue and ANTs	MCF-7, MCF-7/PTX	TPD52, Wnt/β-catenin, Cyclin D1	miR-107, by targeting TPD52 through Wnt/β-catenin signaling pathway, could enhance paclitaxel sensitivity in breast cancer cells	(127)
hsa-miR-125a-3p	Down	37 pairs of BC tissue and normal adjacent tissue with or without doxorubicin treatment	MECs, MCF-7, MCF-7/LCC2, MDA-MB-468, MDA-MB-231, MDA-MB-468/R, MCF-7/R, MDA-MB-468/S, MCF-7/S	BRCA1	hsa-miR-125a-3p, by regulating BRCA1 signaling, could function as a tumor suppressor in breast cancer	(128)
miR-124-3p	Up	40 pairs of BC tissue and ANTs	MCF-7, MCF-7-ADR, MCF-10A, 293T	ABCC4, P-gp	Overexpression of miR-124-3p and downregulation of ABCC4 could increase sensitivity to ADR in MCF-7-ADR cells	(129)
miR-125a	Up	Nude mice	MDA-MB-231, MCF-7, SKBR-3, Hs578T, BT-549, 293T	HER2	miR-125a, by inducing HER2, could enhance the sensitivity to trastuzumab in triple-negative breast cancer cells	(130)
miR-135b-5p	–	28 pairs of BC tissue and ANTs, nude mice	MCF-7, MCF-7/DOXR, MDA-MB-231,	AGR2, Caspase-2, Bak, Bim, Bcl-2, Bcl-xL, Mcl-1	miR-135b-5p, by targeting anterior gradient 2, could enhance the doxorubicin sensitivity of breast cancer cells	(129)
miR-144	–	–	MDA-MB-231, SKBR3	Bax, Bcl-2, N-Cadherin, Vimentin, Snail, AKT, PTEN	miR-144, by targeting PTEN/Akt signaling pathway, could decrease the expression of PTEN and increase the expression of pAKT in MDA-MB-231 and SKBR3 in breast cancer cells	(131)
miR-181a	–	Nude mice	MCF-7, MCF-7/MX	MRP, PGP, LRP, BCRP	miR-181a, by targeting breast cancer resistance protein (BCRP/ABCG2), could enhance drug sensitivity in mitoxantone-resistant breast cancer cells	(132)
miR-181b-2-3p	–	Nude mice	MDA-MB-231, MDA-MB-231/ADR, 293 T	Caspase-3, ABCC3	Curcumol, *via* regulating the miR-181b-2-3p/ABCC3 axis, could enhance the sensitivity to doxorubicin in triple-negative breast cancer	(133)
miR−187−3p	Down	30 pairs of BC tissue and ANTs	MDA-MB-231	FGF9	miR−187−3p, by targeting FGF9 expression, could increase gemcitabine sensitivity in breast cancer cells	(134)
miR-190	–	Nude mice treatment with or without tamoxifen	MCF7, T47D, MDA-MB-453, MDA-MB-468, MDA-MB-231, MDA-MB-435	SOX9, Oct-4, Nanog, ERα, ZEB1, Wnt/β-catenin, c-Myc, Histone-3, TCF4, Cyclin-D1	miR-190, by regulating SOX9 expression, could enhance the sensitivity to endocrine therapy in breast cancer	(135)
miR-195	Down	17 pairs of BC and ANTs	MCF-7, MCF-7/ADR	Raf-1, Bcl-2, P-glycoprotein	Upregulation of miR-195, through inhibition of Raf-1, could increase the sensitivity of breast cancer cells to adriamycin treatment	(136)
miR−205−5p	Down	25 pairs of BC tissue and ANTs	MDA-MB-231, MDA-MB-231/GEM, BT549, MCF10A	ERp29, HSP27	miR−205−5p downregulation by ERp29 upregulation could decrease the gemcitabine sensitivity of breast cancer cells	(137)
miR302a/b/c/d	–	Nude mice	MCF-7, MCF-7/MX	BCRP	miR-302a/b/c/d, through the suppression of BCRP, could increase drug sensitivity in breast cancer cells	(138)
miR-302b	–	–	MDA-MB-231, BT549, T47D	Caspase-3, PARP, E2F, vinculin, ATM	miR-302b, by regulating E2F1 and the cellular DNA damage response, could enhance breast cancer cell sensitivity to cisplatin	(139)
miR-378a-3p	Down	56 pairs of BC tissue and ANTs, Omnibus database	MCF-7, 293T	GOLT1A	miR-378a-3p modulates tamoxifen sensitivity in breast cancer MCF-7 cells through targeting GOLT1A	(140)
miR-381	–	Nude mice	MCF-7, MCF/DOX, MDA-MB-231, MDA-MB-231/DOX	FYN, ERK, p38	miR-381, by inactivation of MAPK signaling *via* FYN, could induce the sensitivity of breast cancer cells to doxorubicin.	(141)
miR-638	–	78 pairs of BC tissue and ANTs	T47D, MCF-7, MDA-MB-231, MDA-MB-468	STARD10	miR-638, *via* regulating STARD10, could lead to potentiation of docetaxel sensitivity in BC cells	(142)
miR-638	–	–	MDA-MB-231, Hs578T, MCF-7, T47D, MCF-10A	BRCA1	miR-638, by regulating BRCA1 expression *via* DNA repair pathways, could enhance radiation and chemotherapy sensitivity in TNBC cells	(143)
miR−1207−5p	Up	30 pairs of TNBC and ANTs with or without paclitaxel treatment	MDA-MB-231, MDA-MB-436, MDA-MB-453, MCF-10A, MDA-MB-293	LZTS1, Bax, Bcl-2, Akt	miR−1207−5p, by suppression of LZTS1 expression, could regulate the sensitivity of triple−negative breast cancer cells to paclitaxel treatment	(144)

ANTs, adjacent normal tissues; PTX, paclitaxel; CDDP, cisplatin.

## Discussion

Non-coding RNAs can influence the expression of several groups of mRNAs through different mechanisms, such as modulation of chromatin structure as well as regulation of transcription and translation. miRNAs are mostly exerting their regulatory roles at the post-transcriptional level through binding to different regions of mRNAs to suppress their translation *via* mRNA degradation or translation inhibition. miRNAs have been found to regulate important aspects of breast carcinogenesis through the regulation of apoptosis, autophagy, and EMT. miRNAs affect the apoptosis of breast cancer cells through several mechanisms; among them are modulation of p53-related pathways, expression of caspases, and regulation of response to ROS. Through modulating the expression of EMT-related genes as well as those influencing cell motility and invasiveness, miRNAs regulate breast cancer metastasis. Notably, miRNAs can also influence the response of breast cancer cells to a wide array of therapeutic agents ranging from conventional chemotherapeutic drugs to tyrosine kinase inhibitors and hormone therapy agents. Based on *in vitro* experiments, miRNAs can regulate the cytotoxic effects of adriamycin, cisplatin, doxorubicin, docetaxel, paclitaxel, gemcitabine, trastuzumab, saracatinib, sunitinib, tamoxifen, and a number of other anti-cancer drugs. In addition to miRNAs whose direct effects on the modulation of response to therapeutic agents have been verified, other miRNAs that regulate cell apoptosis or autophagy can potentially influence therapeutic responses. The modulation of cellular DNA damage response and the activity of cancer stem cells are other routes of participation of miRNAs in the regulation of response of breast cancer cells to chemotherapy. A possible role of miRNAs in the determination of breast cancer stem cells has been suggested through the demonstration of differential expression of miRNAs in CD44+/CD24-/low breast cancer stem cells *versus* non-tumorigenic cancer cells (145). This kind of function of miRNAs has a practical significance in the determination of the behavior of breast cancer as well as its response to therapeutic modalities. In addition, a number of anti-cancer agents exert their effects through the modulation of the expression of miRNAs that affect apoptosis or autophagy—for instance, curcumol, *via* regulating the miR-181b-2-3p/ABCC3 axis, could enhance the sensitivity of triple-negative breast cancer cells to doxorubicin (133). Some miRNAs can affect several aspects of breast carcinogenesis—for instance, miR-34a can affect apoptosis, EMT, and drug resistance. miR-23a has an essential role in the regulation of apoptosis and EMT. Moreover, miR-15a and miR-16a regulate apoptosis and drug resistance. NF-κB, mTOR, and Wnt/β-catenin pathways are among the shared pathways between several miRNAs acting on these processes. Since miRNAs can target multiple transcripts, they can often modulate numerous pathways. Notably, miRNAs exert their inhibitory roles *via* a complex process which is dependent on cellular constituents, indicating tissue or cell type-specific features (146).

The small molecular size of miRNAs and their capacity in the regulation of the expression of genes participating in the evolution of cancer have endowed miRNAs the potential to influence the treatment of breast cancer (147). As miRNAs can affect both the development of breast cancer and the response of cancerous cells to therapeutic options, intervention with their expression is regarded as an appropriate treatment modality for almost every stage of cancer development and progression. Forced over-expression or suppression of miRNA expression is a possible therapeutic modality for breast cancer. Examples of miRNA-antagonism methods are 2′-O-methyl-modified oligonucleotides, locked nucleic acid anti-miRNAs, and cholesterol-conjugated antagomirs. These methods are being used as miRNA-inhibitory tools (148). In fact, the over-expression of miRNAs that induce cell apoptosis, such as miR-7-5p (16), miR-15a, miR-16 (19), and miR-17-5p (20), or inhibit cell cycle progression can suppress the progression of breast cancer. On the other hand, the suppression of expression of oncogenic miRNAs by oligo antisense mechanisms is a treatment modality. *In vitro* studies have provided a firm evidence for the specificity and efficacy of miRNA-based modalities in the modulation of the expression of target genes, yet future studies should focus on the improvement of delivery systems, enhancement of stability of the prescribed molecules, decreasing off-target effects, and assessment of long-term safety of these molecules (149). Only after solving these issues can miRNA-based therapeutics enter clinical practice.

The identification of the miRNA-associated network and interplay between miRNAs and other types of regulatory transcripts will open new opportunities for diagnostics and therapeutic modalities in breast cancer. System biology methods can be used to predict the role of miRNAs in the determination of response to anti-cancer therapies and prognostic approaches in clinical settings. Targeting miRNAs with essential roles in a drug-resistant network has been suggested as a putative approach in overcoming chemoresistance in breast cancer (146). Finally, the combinations of conventional anticancer drugs with anti-oncogenic miRNA reagents are expected to enhance treatment responses. In fact, the recognition of miRNA profiles in different stages of breast cancer development and development of miRNA-based targeted therapies are two wings of miRNA studies which can introduce novel promising results in clinical settings.

In brief, the contribution of miRNAs in the regulation of cell death, cell motility and invasion, activity of cancer stem cells, regulation of EMT, and modulation of response to therapeutics potentiate miRNAs as proper targets for the treatment of breast cancer. However, the clinical application of miRNA-based therapies depends on the effective documentation of miRNA profiles in different subtypes of breast cancer and the construction of the interaction network between miRNAs and genes that regulate breast carcinogenesis and chemoresistance phenotype.

## Author Contributions

SG-F wrote the draft and revised it. MT designed and supervised the study. AK, AA, HS, and AS collected the data, designed the figures and tables. All the authors read and approved the submitted version.

## Conflict of Interest

The authors declare that the research was conducted in the absence of any commercial or financial relationships that could be construed as a potential conflict of interest.

## Publisher’s Note

All claims expressed in this article are solely those of the authors and do not necessarily represent those of their affiliated organizations, or those of the publisher, the editors and the reviewers. Any product that may be evaluated in this article, or claim that may be made by its manufacturer, is not guaranteed or endorsed by the publisher.
